# SGLT2 Inhibitors Between Benefits and Euglycemic Ketoacidosis: A Concise Review

**DOI:** 10.3390/ijms27125224

**Published:** 2026-06-09

**Authors:** Luminita-Georgeta Confederat, Alin-Constantin Pînzariu, Ionela Lacramioara Serban, Mihaela-Iustina Condurache, Oana-Maria Dragostin

**Affiliations:** 1 Grigore T. Popa University of Medicine and Pharmacy Iasi, 700115 Iasi, Romania; alin.pinzariu@umfiasi.ro (A.-C.P.); ionela.serban@umfiasi.ro (I.L.S.); mihaela-iustina.condurache@umfiasi.ro (M.-I.C.); 2 “Sfântul Spiridon” County Emergency Clinical Hospital, 700111 Iasi, Romania; 3 Research Centre in the Medical-Pharmaceutical Field, Faculty of Medicine and Pharmacy, “Dunarea de Jos” University of Galati, 800008 Galati, Romania; oana.dragostin@ugal.ro

**Keywords:** SGLT2 inhibitors, benefits, euglycemic ketoacidosis, risk factors, management

## Abstract

Diabetes mellitus is a complex metabolic disorder whose management has moved from glycemic control to the control of risk factors through the use of new antihyperglycemic drugs with pleiotropic effects. Despite the multiple cardio–renal benefits of sodium-glucose co-transporter 2 (SGLT2) inhibitors, their prescription is often avoided due to concerns regarding side effects. This review aims to discuss the multiple benefits of SGLT2 inhibitors in balance with one of the most concerning side effects, the risk of euglycemic diabetic ketoacidosis (EDKA). A literature search was performed to identify and select articles relevant to this topic. We accessed several databases, including PubMed, Web of Science and Scopus, using appropriate keywords. We selected and evaluated randomized controlled trials, retrospective studies, systematic reviews and meta-analysis published between 2014 and 2024 supporting the multifaceted benefits of SGLT2 inhibitors and the limitations of their recommendations and focusing on the risk of EDKA. Initially designed as antidiabetic agents, SGLT2 inhibitors have demonstrated important cardio–renal benefits, these drugs being the first-line medication in patients with established cardiovascular disease, heart failure and chronic kidney disease. SGLT2 inhibitors are associated with some potential side effects, but with contradictory data concerning their prevalence and clinical relevance. From the possible side effects, EDKA is a life-threatening metabolic emergency whose incidence and recognition has increased, in particular with the use of SGLT2 inhibitors. These drugs can cause this disorder through several mechanisms, including reduced insulin secretion and increased glucagon levels, leading to free fatty acid production, which generally occurs in the presence of some risk factors such as reduced dietary carbohydrates, intercurrent illnesses, surgical stress and alcohol consumption. Through awareness of these risk factors as well as of the clinical symptoms, this condition could be promptly avoided or managed and SGLT2 inhibitors could be safely used.

## 1. Introduction

Diabetes mellitus is a complex metabolic disorder whose main characteristic is an impairment of glucose homeostasis, leading to chronic hyperglycemia, as a result of insulin deficiency or insulin resistance [1]. In recent years, diabetes mellitus has become a major public health concern because of the alarming increase in prevalence, the morbidity and mortality associated with its complications and, not least of all, the costs involved in taking care of patients with diabetes [2].

According to International Diabetes Federation (IDF) reports, the prevalence of the disease has notably increased in every region of the world during the last two decades; thus, in 2021 there were 537 million people with diabetes, counting for 10.5% of the global population; if this trend is maintained, the IDF predictions show that by 2030, 643 million people will be affected by this disease, and by 2045, the number of cases will reach 783 million people, meaning 12% of the world population [3].

Poorly controlled hyperglycemia can lead to serious microvascular and macrovascular complications, with reduced life expectancy and life quality [4]. The microvascular complications of diabetes include diabetic retinopathy—the main cause of blindness among patients with diabetes—and diabetic chronic kidney disease, which affects approximately 50% of people with diabetes and contributes to the personal, social and economic burden of the disease [5,6]. Macrovascular complications of diabetes are related to different types of atherosclerotic cardiovascular disease, including ischemic heart disease, stroke and peripheral vascular disease; in addition to this, a consensus report of the American Diabetes Association (ADA) has established heart failure as an underappreciated complication of diabetes that may develop even in the absence of hypertension, dyslipidemia or coronary or valvular heart disease [7,8,9].

As an outcome of this dramatic increase in prevalence, diabetes represents a substantial economic burden to health-care systems. According to the Global Burden of Disease Study 2021, the expenditures related to diabetes care were USD 966 billion globally, and these are expected to reach to USD 1054 billion by 2045 [10,11].

From the above-mentioned chronic complications of diabetes, atherosclerotic cardiovascular disease remains the main cause of morbidity and mortality in patients with diabetes, being 2–4 times more frequent compared to the general population [12].

Considering the progressive nature of the disease, it is widely accepted that achieving good blood glucose control as early as possible can reduce the risk of microvascular and macrovascular complications, including diabetic nephropathy, diabetic retinopathy and cardiovascular disease, as well as mortality attributed to diabetes [13]; at the same time, it has become clear that targeting strict glucose control alone may not be enough in the management of a multifaceted disease like diabetes, and multiple risk factors should be addressed simultaneously in order to reduce the risk [5]. Presently, diabetes care has moved beyond blood glucose values and glycosylated hemoglobin (HbA1c), targeting cardiovascular and renal protection through the control of multiple risk factors and implementation of the use of new antihyperglycemic drugs with pleiotropic effects [14]. Additionally, for several current blood glucose-lowering agents, an optimal glycemic control may be difficult to obtain without clinically relevant side effects such as hypoglycemia and weight gain, being another argument to support the use of new classes of drugs with multiple benefits [15].

In accordance with the dramatic increase in prevalence of diabetes, its devastating complications and the obvious necessity to address the multiple risk factors involved, several studies have been focused on the development of different classes of drugs in order to obtain and maintain an optimal glycemic control, together with cardiovascular protection. The treatment of diabetes mellitus recorded remarkable progress, and at present, there are nine available classes of antihyperglycemic agents. Older antidiabetic drugs, including sulfonylureas, metformin, thiazolidinediones, meglitinides and dipeptidyl peptidase 4 inhibitors, reduced the risk of microvascular complications, but with no significant benefits for atherosclerotic cardiovascular disease or kidney disease [16,17]. The new classes of antidiabetic agents available, glucagon-like peptide 1 receptor agonists (GLP 1-RA) and sodium-glucose co-transporter 2 (SGLT2) inhibitors, have demonstrated cardio–renal benefits beyond glycemic control, a fact that has led to important changes in the latest guidelines recommendations of American Diabetes Association (ADA), these drugs being the first-line of medication for patients with established cardiovascular disease, heart failure and chronic kidney disease [17,18].

The use of new classes of antihyperglycemic drugs with pleiotropic effects is supported by a paradigm shift in the management of diabetes: from a glucose-centered approach to the simultaneous control of multiple risk factors and the reduction of cardiovascular risk. SGLT2 inhibitors are valuable therapeutic agents that have all the arguments to be recommended in the complex management of diabetes mellitus. Nevertheless, their prescription is avoided because of precaution reasons regarding the risk of potential side effects, from which the most concerning is euglycemic diabetic ketoacidosis (EDKA). The main gap in clinical practice is to find the balance between prescribing drugs with multiple demonstrated benefits and finding strategies to reduce the risk of side effects, an aspect that will be discussed within this paper.

This review will be focused on SGLT2 inhibitors, discussing the multiple cardio–reno–metabolic benefits in balance with one of the most concerning and life-threatening side effects, the risk of euglycemic diabetic ketoacidosis.

A literature search was performed to identify and select articles related to the demonstrated benefits of SGLT2 inhibitors and EDKA development in patients being treated with this class of drug. We accessed several databases, including PubMed, Web of Science and Scopus, to search for published literature on this topic. The keywords used included but were not limited to “sodium-glucose transporter 2 inhibitors benefits”, “SGLT2-inhibitors cardiovascular/renal benefits”, “euglycemic diabetic ketoacidosis”, “EDKA and SGLT2-inhibitors” and “EDKA risk factors”. We evaluated randomized controlled trials, retrospective studies, systematic reviews and meta-analysis published between 2014 and 2024. A literature search of the references cited in the identified publications was conducted and a few older references were included only if these were relevant to the objective of the study. Published guidelines were also reviewed. The exclusion criteria consisted of publications not relevant to the aim of the review, abstracts without full text available and articles in other languages than English.

## 2. SGLT2 Inhibitors

Initially designed as antidiabetic agents, SGLT2 inhibitors are drugs that have demonstrated multiple benefits beyond the antihyperglycemic action, such as cardiovascular and renal protection, weight loss, decrease of blood pressure, improvement of lipid profile, benefits for non-alcoholic fatty liver disease and the modulation of endothelial function and arterial stiffness. Currently, there are four oral drugs from this therapeutic class approved in diabetes treatment (canagliflozin, dapagliflozin, empagliflozin and ertugliflozin), with different selectivity and potency against SGLT2 [19]. This section will address the mechanism of action and the main demonstrated benefits and side effects of SGLT2 inhibitors.

### 2.1. Mechanism of Action

Under normal physiological conditions, the glucose filtered is nearly entirely reabsorbed at the kidney level via SGLT2 (responsible for almost 90% of glucose reabsorption) and SGLT1 (responsible for the remaining 10%); thus, glucose is not present in the final urine. SGLT2 inhibitors’ mechanism of action consists of the competitive inhibition of SGLT2 receptors, reducing the reabsorption of glucose in the proximal kidney tubule and inducing sustained glycosuria [20]. When blood glucose values exceed 180 mg/dL, the value considered the threshold for glycosuria, the filtered glucose is excreted in the urine. In addition to this, in hyperglycemic conditions, the expression of SGLT2 receptors is up-regulated, with this phenomenon being responsible for excessive glucose reabsorption and perpetuating maladaptive hyperglycemia. The glucose-lowering effect of SGLT2 inhibitors is proportional to the amount of filtered glucose, meaning that the efficacy of this class of drugs is higher in patients with higher glycemic values. Finally, this mechanism of action is independent of insulin, so the risk of hypoglycemia is minimal; nevertheless, the association of SGLT2 inhibitors with insulin or insulin secretagogues could increase this risk [20,21].

### 2.2. Glycemic Control

Several trials with SGLT2 inhibitors were focused on glycemic control in treatment-naive patients or when added to different classes of antidiabetic drugs; the results demonstrated their effectiveness assessed through fasting plasma glucose, postprandial glucose levels and glycosylated hemoglobin (HbA1c) values [22]. Dapagliflozin demonstrated a reduction of HbA1c levels by 0.82–0.89% after 24 weeks of administration in treatment-naive patients and by 0.84% when added to metformin, with higher reductions being observed in patients with higher HbA1c values at baseline [23]. Additionally, dapagliflozin reduced HbA1c levels by 0.82% in patients treated with glimepiride, by 0.97% when added to pioglitazone and by 0.57% when added to basal insulin [24,25,26]. Canagliflozin reduced HbA1c levels by 1.03% after 26 weeks of administration in naive-treatment patients with type 2 diabetes, by 0.74% in those treated with metformin and by 0.97% in patients inadequately controlled with basal insulin, diet and physical exercise [27,28,29]. Similarly, empagliflozin demonstrated a HbA1c reduction of 0.85% after 24 weeks of treatment in monotherapy, 0.77% when added to metformin and 0.7% in patients on inadequately controlled basal insulin [22,30].

On the other hand, randomized controlled trials and real-world studies were carried out that evaluated the glycemic benefits of SGLT2 inhibitors in patients with type 1 diabetes. A meta-analysis conducted by Li et al. highlighted that SGLT2 inhibitors reduced HbA1c values by 0.47% compared to a placebo, with higher doses being associated with greater reduction, suggesting a dose-dependent effect [31,32]. There were controversial results related to the fact that certain groups of individuals are more likely to obtain a greater HbA1c reduction; in this regard, the EASE trials and a real-world study evidenced that higher HbA1c reduction was achieved by patients with a baseline HbA1c > 8% and body mass index > 27 kg/m^2^ [33]. By contrast, the DEPICT-1 study showed no significant differences in glycemic benefits related to baseline HbA1c and body mass index [34]. Concerning other parameters indicating glycemic control (fasting plasma glucose, mean daily glucose, time in range, post-prandial glucose levels and glycemic variability), the results of the studies showed that fasting plasma glucose and mean daily glucose were reduced dose-dependently, while the percentage time-in-range increased by approximately 14% with SGLT2 inhibitors compared to a placebo [35]; additionally, the time in the hyperglycemic range decreased in a dose-independent manner without an increase in the risk of hypoglycemia [32].

### 2.3. Cardiovascular Benefits

Considering the fact that cardiovascular disease is the most frequent comorbidity in patients with type 2 diabetes and represents the leading cause of mortality among these individuals, there were several trials carried out with cardiovascular outcomes in order to demonstrate the cardiovascular safety or cardiovascular protection of antidiabetic drugs.

The EMPA-REG OUTCOME trial evaluated the cardiovascular safety profile of empagliflozin versus a placebo in about 7000 patients with type 2 diabetes and established cardiovascular disease (coronary, cerebrovascular or peripheral arterial disease); the results evidenced a reduction in death from cardiovascular causes by 38%, death from any cause by 32% and hospitalization for heart failure by 35%, while there was no influence on myocardial infarction and stoke [36]. The DECLARE-TIMI trial studied the influence of dapagliflozin on cardiovascular events in about 17,000 individuals with type 2 diabetes and established atherosclerotic cardiovascular disease or multiple risk factors, randomized to receive dapagliflozin or placebo; the findings showed that SGLT2 inhibitor dapagliflozin reduced cardiovascular death and hospitalization for heart failure by 17% [37]. Similarly, a CANVAS study compared canagliflozin with a placebo and demonstrated a reduction by 14% of the composite 3P-MACE (death from cardiovascular causes, non-fatal myocardial infarction and non-fatal stroke) [38].

Following the remarkable results of SGLT2 inhibitors within cardiovascular outcome trials, there were preclinical and clinical studies carried out that demonstrated multiple extra-glycemic mechanisms, which might explain the cardiovascular benefits of these drugs. SGLT2 induces osmotic diuresis, promoting the excretion of electrolyte-free water compared with conventional diuretics, allowing for a higher reduction of interstitial fluid than intravascular and thus improving tissue congestion with the preservation of tissue perfusion [39]. Additionally, these drugs reduced arterial stiffness and blood pressure by 3–5 mmHg, having a positive effect both on cardiac pre-load and post-load; furthermore, SGLT2 inhibitors could positively modulate cardiac metabolism by promoting the use of substrates as ketone bodies and glucose, with more favorable adenosine triphosphate (ATP) to oxygen ratios. Finally, there are studies that sustain the benefits of SGLT2 inhibitors on inflammation, oxidative stress, cardiomyocytes contractility, cardiac remodeling and ischemia-reperfusion injuries, with all of these effects contributing to cardiovascular protection [40].

### 2.4. Renal Benefits

In terms of renal protection, there were several randomized controlled trials with renal outcomes that demonstrated the efficacy of SGLT2 inhibitors in slowing the progression of diabetic kidney disease. The CREDENCE trial evaluated the renal effects of canagliflozin versus a placebo in patients with type 2 diabetes with macroalbuminuria and estimated glomerular filtration rate (eGFR) between 30 and 90 mL/min/1.73 m^2^; the results demonstrated a 32% risk reduction in the composite endpoint of end-stage kidney disease, two-fold increase in serum creatinine, renal or cardiovascular death [41]. Similarly, the DAPA-CKD study included about 4300 patients with chronic kidney disease, two-thirds of them with diabetes and one-third without diabetes, treated with dapagliflozin versus a placebo; the results extended these renal benefits to a broader population, irrespective of diabetes status, showing a 39% reduction in the risk of the composite renal outcome [42]. Finally, empagliflozin demonstrated a relative risk reduction of 28% in the progression of kidney disease in the EMPAREG OUTCOME trial [18].

The mechanisms proposed for SGLT2-mediated renal protection are multifactorial. SGLT2 inhibitors increase the delivery of sodium to the macula densa, restoring tubulo-glomerular feedback and thus being able to revert hyperglycemia-induced glomerular hyperfiltration in diabetic kidneys [43]. These drugs induce osmotic diuresis and natriuresis, preserving intravascular volume and reducing interstitial overload, also contributing to optimal renal perfusion [43]. The benefits of SGLT2 inhibitors on the kidney could also be due to the reduction of serum uric acid levels, as hyperuricemia has been associated with glomerular hypertension, interstitial fibrosis and the loss of tubular cell viability [44]. Additionally, experimental studies showed anti-inflammatory effects for these drugs by down-regulating pro-inflammatory cytokines such as interleukin-6 and tumor necrosis factor-alpha in kidneys; also, SGLT2 inhibitors have antifibrotic action by inhibiting pro-fibrotic mediators and reducing the deposition of extracellular matrix, thus preserving renal function [45,46].

### 2.5. Other Benefits

In addition to glycemic control and cardiovascular and renal protection demonstrated by extensive randomized controlled trials, SGLT2 inhibitors have demonstrated other important benefits evidenced in experimental models or clinical observations.

#### 2.5.1. Weight Control

SGLT2 inhibitors are known to induce modest weight loss, typically between 2 and 4 kg, resulting from a caloric deficit created by increased urinary excretion of glucose, which can range between 70 g to 100 g daily. However, the magnitude of this effect is limited by compensatory mechanisms and adaptative metabolic responses such as increased appetite, decreased resting energy expenditure and alterations in substrate utilization, leading to the limitation of the weight loss obtained with these drugs [20,47].

#### 2.5.2. Adipose Tissue Inflammation and Activation

The effect of SGLT2 inhibitors on adipose tissue is not only limited to weight loss; these drugs exert multifaceted effects on adipose tissue, influencing tissue remodeling and metabolic health through different mechanisms. Firstly, SGLT2 inhibitors promoted a phenotypic switch from proinflammatory M1 macrophages to anti-inflammatory M2 macrophages, thus reducing the secretion of proinflammatory cytokines and improving adipose tissue function [20,48]. Secondly, these agents sustained the browning of white adipose tissue, a process that is associated with increased energy expenditure and improved metabolic flexibility [49]. Finally, experimental models suggested that SGLT2 inhibitors may activate brown adipose tissue, a phenomenon associated with increased thermogenesis and lipid oxidation [50]. All these mechanisms involving the induction of anti-inflammatory phenotypes in adipose tissue, enhancement of thermogenic capacity and facilitation of metabolic improvements contribute together to the important role of SGLT2 inhibitors in managing obesity and other metabolic disturbances associated.

#### 2.5.3. Non-Alcoholic Fatty Liver Disease

A systematic review and network meta-analysis of randomized controlled trials included patients with non-alcoholic fatty liver disease, with or without type 2 diabetes treated with SGLT2 inhibitors compared with placebo/standard treatments. The outcomes included hepatic cytolysis enzymes and gamma-glutamyl transferase levels, fibrosis-4 score, body weight and fasting plasma glucose values. The results showed that SGLT2 inhibitors significantly improved liver enzymes, fibrosis-4 score and metabolic outcomes in patients with non-alcoholic fatty liver disease. Dapagliflozin was superior in improving liver enzymes and body weight, while empagliflozin was proven to have a greater effect on fibrosis markers, with all these findings supporting personalized treatment regimens, depending on the predominant disturbance [51]. Moreover, the ongoing ENLIGHTENED trials suggest the synergistic potential of the association between empagliflozin and semaglutide, which showed a preliminary non-alcoholic steatohepatitis resolution rate of 62% [52].

#### 2.5.4. Polycystic Ovary Syndrome

A meta-analysis conducted in 2024 by Javed et al. found that SGLT2 inhibitors improved hyperandrogenism and reduced free testosterone levels by 1.8 pg/mL [53].

#### 2.5.5. Neurodegenerative Diseases

The link between ketone metabolism and neuroprotection was addressed in some ongoing studies such as Alzheimer’s EMPA-REG-NEURO trial, in which the administration of 25 mg empagliflozin for 18 months showed a 1.8-point ADAS-Cog14 improvement and also a favorable change in the composition of cerebrospinal fluid [54].

#### 2.5.6. Cancer Treatment

In addition to their established physiological function in kidney proximal tubules, SGLT2 has been identified in specific tumor cells, a fact that raised the hypothesis related to their potential as therapeutic targets. Considering the fact that many cancer cells change their metabolism to be more glucose-dependent, reducing glucose reabsorption with SGLT2 inhibitors may limit this mechanism of tumor growth. Several drugs from this class, such as dapagliflozin, canagliflozin and empagliflozin, have been shown in experimental studies to cause apoptosis, decrease the growth of many cancer cell types and alter important cellular signaling pathways [1].

### 2.6. Side Effects

Although SGLT2 inhibitors have multiple indications related to their proven metabolic, cardiovascular and renal benefits, this class of drugs is known to cause certain side effects, some of them being the result of their mechanism of action and other reported cases. The adverse events associated with SGLT2 inhibitors include diabetic ketoacidosis (DKA), euglycemic diabetic ketoacidosis (EDKA), genital infections, urinary tract infections, volume depletion and hypotension, risk of fracture, lower limb amputation and Fournier’s gangrene [55,56].

The data concerning clinical relevance and prevalence of these side effects are contradictory. According to the analysis conducted by Zhou et al., from the above-mentioned side effects, DKA, EDKA and Fournier’s gangrene were more likely to occur when using SGLT2 inhibitors compared to other antidiabetic drugs [57]. A meta-analysis studied all four SGLT2 inhibitors in type 2 diabetes, heart failure and chronic kidney disease; the results showed that these drugs significantly increased the risk for DKA, genital infections, urinary tract infections, volume depletion, limb amputation and fractures [55].

**Figure 1:**
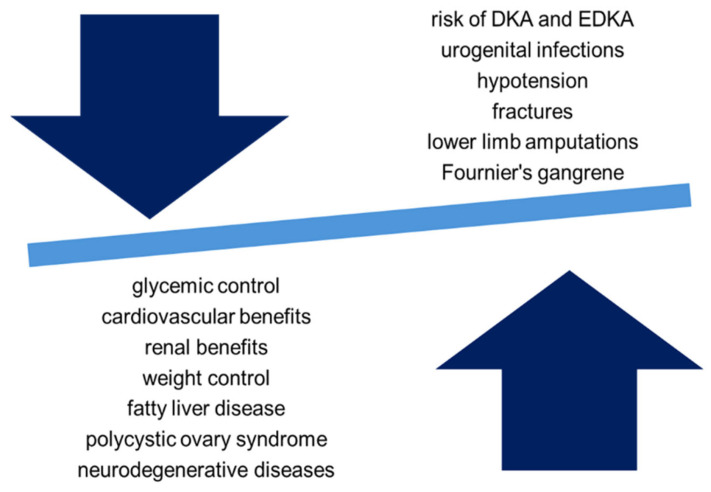
Benefits and side effects of sodium-glucose co-transporter 2 (SGLT2) inhibitors.

SGLT2 inhibitors may cause hypovolemia and hypotension as a result of their mechanism of action; natriuresis and osmotic diuresis lead to a decrease in extracellular volume and to the activation of the renin–angiotensin system, resulting in the activation of angiotensin-2 receptors followed by vasodilatation [58]. DKA is explained by the indirect action of these drugs that increase glucagon secretion and decrease serum insulin levels; additionally, they increase ketogenesis and the renal absorption of ketones [59]. EDKA is a severe complication of SGLT2 inhibitors that can mask the signs and symptoms of DKA, triggered by factors such as severe insulin deficiency, a low-carbohydrate diet, dehydration, alcohol intake and some severe diseases [57,60]. Urogenital infections can be explained by increased glucose concentrations in the urinary tract and gender-dependent factors, while the cause for amputations could be the decreased peripheral perfusion as a result of extracellular volume reduction [60]. An extended pharmacovigilance study compared the side effects reported in randomized controlled trials with SGLT2 inhibitors with real-world reported data. The results showed differences in the reported adverse effects for different drugs from this class between clinical trials and real world-data. For empagliflozin, urinary tract infections and hypotension were the most common adverse effects in clinical trials, while DKA was the most frequently reported in real-world data. Concerning dapagliflozin, clinical trials reported urinary tract infections, amputations and hypotension, while databases found DKA and amputations to be the most common side effects. For canagliflozin, there were urogenital infections from clinical trials and DKA and amputations from real-world databases [61]. The balance between the benefits and side effects of SGLT2 inhibitors is illustrated in Figure 1.

## 3. Euglycemic Ketoacidosis

Diabetic ketoacidosis (DKA) is an acute life-threatening metabolic emergency that is defined by three main factors: hyperglycemia, metabolic acidosis with increased anion gap and ketosis [62]. Even if this acute complication occurs more frequently in patients with type 1 diabetes, up to 23% of cases are reported in patients with type 2 diabetes [63]. Euglycemic diabetic ketoacidosis (EDKA) is a subset of DKA characterized by relative euglycemia (blood glucose value < 250 mg/dL), metabolic acidosis (serum bicarbonate < 18 mEq/L and pH < 7.3) and ketosis [64]. In recent years, the incidence and recognition of this condition have increased, in particular with the use of SGLT2 inhibitors, with this class of drugs being associated with a 7-fold higher risk of EDKA. Additionally, DKA in insulin-dependent patients with diabetes may be due to the SGLT2 inhibitors in 5–12% of cases, while for DKA admissions, 2.6–7% of patients present the euglycemic form [65].

### 3.1. Pathophysiology

The pathophysiology of EDKA involves absolute insulin deficiency or relative insulin deficiency in the presence of severe insulin resistance; this condition is associated with stimulation of glucagon production and increased release of free fatty acids, leading to ketogenesis and acidosis. The underlying mechanism of EDKA also includes reduced glucose availability and secretion associated with stressors and/or increased urinary excretion of glucose associated with the excessive production of counter-regulatory hormones [66,67]. Consequently, any condition leading to decreased glucose availability or production, reduced insulin secretion or increased counter-regulatory hormones level could cause EDKA.

**Figure 2:**
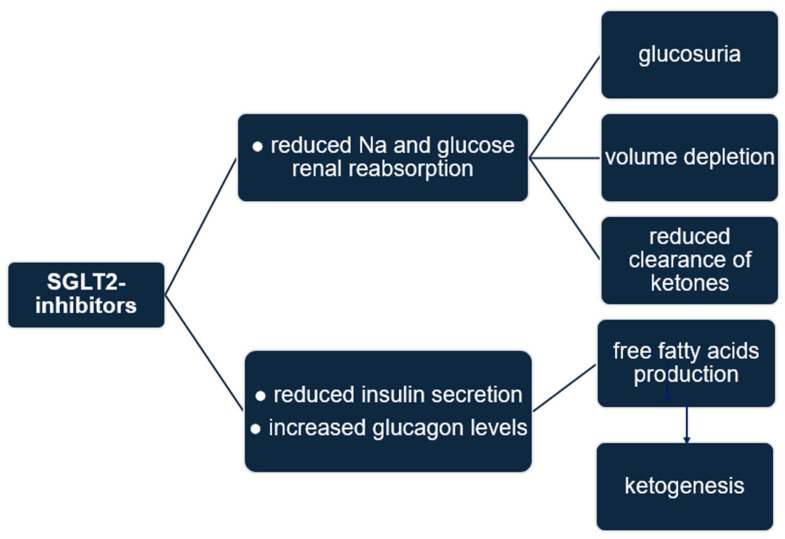
Pathophysiology of euglycemic diabetic ketoacidosis (EDKA).

EDKA associated with the administration of SGLT2 inhibitors can be explained by several mechanisms, including reduced insulin secretion and increased glucagon levels, leading to free fatty acid production, which generally occurs in the presence of some stressors such as infection, ischemia and starvation, even if the exact etiology is not identified in all EDKA cases [63,68]. As mentioned above, SGLT2 inhibitors act on the corresponding cotransporter protein situated in the proximal renal tubules, which is responsible for the reabsorption of about 90% of filtered glucose. These drugs block the reabsorption of glucose and increase its urinary excretion, resulting in glycosuria, reduced serum availability of glucose, volume depletion and reduced clearance of ketones, explaining blood glucose values lower than in other cases of DKA [63]. The pathophysiology of EDKA is illustrated in Figure 2. According to the literature data, the typical moment of the onset of EDKA is less than two months after the initiation of SGLT2 inhibitors, and canagliflozin was associated with the highest risk [69].

### 3.2. Risk Factors

The above-mentioned mechanism does not cause EDKA in the majority of patients with diabetes treated with SGLT2 inhibitors, but these changes in systemic metabolism create a susceptibility state for the development of this complication in the presence of some risk factors. Table 1 summarizes the main risk factors for SGLT2 inhibitor-associated EDKA.

### 3.3. Clinical Manifestations

The clinical presentation of EDKA is similar with DKA, but due to the relatively lower blood glucose level, about 50% of patients are diagnosed with delay. The symptoms experienced by patients with EDKA include nausea and vomiting, abdominal pain and fatigue; Kussmaul breathing and different degrees of alteration of mental status may occur, depending on the degree of acidosis [71,72]. Patients are commonly dehydrated and may present with tachycardia, hypotension, dry mucous membranes, delayed capillary refill and poor skin turgor [71]. A review of 72 cases of EDKA associated with SGLT2 inhibitors showed that the most common symptoms experienced by patients were nausea (48%), abdominal pain (38%) and vomiting (36%), while the most frequent signs were tachypnoea (34%), tachycardia (30%), dehydration (14%) and altered mental status (10%) [73].

### 3.4. Management

The immediate management of EDKA represents an emergency and is based on three main aspects. The first aspect is initial fluid resuscitation with 1–2 L during the first 1–2 h; balanced crystalloids are recommended in order to avoid hyperchloremic non-anion gap acidosis caused by normal saline solution. Secondly, the initiation of insulin at a rate of 0.05–0.1 units/kg/h is recommended for the control of ketosis in patients with a serum potassium level > 3.5 mEq/L. In order to avoid hypoglycemia, dextrose 5% or 10% should be administered concurrently with intravenous insulin. Finally, the serum level of potassium should be controlled; potassium supplementation is recommended in patients with serum values between 3.5 and 5.5 mEq/L; if its value is >5.5 mEq/L, the supplementation should be avoided, and at values < 3.5 mEq/L, the potassium level should be corrected before initiating insulin infusion [63,66,74].

## 4. Conclusions

Initially designed as antidiabetic agents, SGLT2 inhibitors have demonstrated multifaceted benefits beyond glycemic control, as shown in large randomized controlled trials with cardiovascular or renal outcomes, real-world studies or experimental models. In addition to the extensively studied cardiovascular and renal protection that changed the latest recommendations of the current guidelines, these drugs have shown benefits in weight management, systemic inflammation and non-alcoholic fatty liver disease, as well as promising results in polycystic ovary syndrome, neurodegenerative diseases and even different types of cancers. The results of these studies evidenced differences in the magnitude of these mentioned benefits between the drugs belonging to this class, supporting personalized treatment regimens. The side effects associated with SGLT2 inhibitors could be explained by their mechanism of action, and these are generally triggered or favored by some risk factors that could, at least partially, be controlled. However, data concerning the clinical relevance and prevalence of these side effects are contradictory. From the extensive list of possible side effects, euglycemic diabetic ketoacidosis is a metabolic emergency that requires prompt diagnosis and management. Thus, clinicians should be aware of the risk factors that could precipitate it, and also, they should educate patients concerning symptoms in order to avoid this acute complication. Finally, SGLT2 inhibitors remain valuable therapeutic agents that can be used safely, and, if the risk factors for precipitating side effects are controlled, their pleiotropic benefits could be maximized.

## Figures and Tables

**Table 1:** Risk factors for sodium-glucose co-transporter 2 (SGLT2) inhibitors-associated euglycemic diabetic ketoacidosis (EDKA) [70].

Risk Factor	Mechanism	Precautions
Reduction in insulin dose	Stimulation of lipolysis and increased glucagon–insulin ratio predisposing to ketogenesis in liver	Avoid reduction in insulin dose > 20% Monitoring urine ketones following insulin dose adjustments
Restriction in dietary carbohydrates availability	Increased lipolysis leads to increased glucagon–insulin ratio and ketogenesis	Avoid very low-carbohydrate diets
Surgical stress, trauma, intercurrent illness	Decreased food intake, leading to reduced insulin levels and ketogenesis Increased level of counter-regulatory hormones; accelerate lipolysis	Discontinue SGLT2-i prior to elective surgery and with acute illness
Alcohol intake	Increased lipolysis and decreased insulin–glucagon ratio leading to ketogenesis	Avoid excessive alcohol intake/be aware of this situation

## Data Availability

No new data were created or analyzed in this study. Data sharing is not applicable to this article.
